# Network-based analysis of differential white matter connectivity in major depressive disorder with and without comorbid anxiety

**DOI:** 10.1038/s41386-025-02312-y

**Published:** 2026-01-12

**Authors:** Marius Gruber, Jan Schulte, Marco Mauritz, Kira F. Ahrens, Paula Rehm, Nina M. von Werthern, Henry Staub, Stefanie Fischer, Franka Timm, Ilan Libedinsky, Pascal Grumbach, Linda M. Bonnekoh, Janik Goltermann, Nils Ralf Winter, Katharina Thiel, Alexandra Winter, Tiana Borgers, Melissa Klug, Hannah Meinert, Julia Hubbert, Judith Krieger, Christoph Jurischka, Florian Thomas-Odenthal, Paula Usemann, Lea Teutenberg, Marc Pawlitzki, Katharina Förster, Lisa Sindermann, Joscha Böhnlein, Susanne Meinert, Dominik Grotegerd, Frederike Stein, Benjamin Straube, Nina Alexander, Hamidreza Jamalabadi, Andreas Jansen, Igor Nenadić, Nils Opel, Tim Hahn, Jochen Bauer, Martijn P. van den Heuvel, Andreas Reif, Tilo Kircher, Elisabeth J. Leehr, Udo Dannlowski, Jonathan Repple

**Affiliations:** 1 Institute for Translational Psychiatry, University of Münster, Münster, Germany; 2 Goethe University Frankfurt, University Hospital, Department for Psychiatry, Psychosomatic Medicine and Psychotherapy, Frankfurt, Germany; 3 Goethe University Frankfurt, Cooperative Brain Imaging Center - CoBIC, Frankfurt, Germany; 4 Connectome Lab, Department of Complex Trait Genetics, Center for Neurogenomics and Cognitive Research, Vrije Universiteit Amsterdam, Amsterdam Neuroscience, Amsterdam, The Netherlands; 5 Institute of Neurosciences and Medicine, Brain & Behaviour (INM-7), Research Centre Juelich, Juelich, Germany; 6 Department of Child and Adolescent Psychiatry, Psychosomatics and Psychotherapy, University of Münster, Münster, Germany; 7 Department of Psychiatry and Psychotherapy, University of Marburg, Marburg, Germany; 8 Center for Mind, Brain and Behavior, University of Marburg, Marburg, Germany; 9 Department of Neurology, Medical Faculty, Heinrich Heine University Dusseldorf, Düsseldorf, Germany; 10 Clinical Psychology and Behavioral Neuroscience, Faculty of Psychology, Technische Universität Dresden, Dresden, Germany; 11 Child and Adolescent Psychotherapy, Institute of Psychology, Universität Hamburg, Hamburg, Germany; 12 Department of Psychiatry and Psychotherapy, University Hospital Bonn, Bonn, Germany; 13 Clinical Psychology and Translational Psychotherapy, Department of Psychology, University of Münster, Münster, Germany; 14 Institute of Translational Neuroscience, University of Münster, Münster, Germany; 15 Department of Psychiatry & Neuroscience, Campus Benjamin Franklin, Charité Universitätsmedizin Berlin, Berlin, Germany; 16 German Centre for Mental Health (DZPG), Partner Site Berlin-Potsdam, Berlin, Germany; 17 Department of Radiology, University of Münster, Münster, Germany; 18 Department of Child Psychiatry, Amsterdam University Medical Center, Amsterdam Neuroscience, Amsterdam, The Netherlands; 19 Fraunhofer Institute for Translational Medicine and Pharmacology ITMP, Frankfurt am Main, Germany; 20 Department of Psychiatry, Medical School and University Medical Center OWL, Protestant Hospital of the Bethel Foundation, Bielefeld University, Bielefeld, Germany

**Keywords:** Depression, Anxiety

## Abstract

Current psychiatric neuroimaging supports the view that major depressive disorder (MDD) is a dysconnection syndrome, characterized by structural brain dysconnectivity. Recent studies investigating this question, however, did not evaluate the involvement of comorbid disorders, of which anxiety disorders (ANX) are particularly prevalent. Here, we investigated the structural connectivity alterations observed in MDD with and without comorbid ANX. To this end, we reconstructed structural brain networks of *n* = 781 individuals with a diagnosis of MDD who had at least one diagnosis of an ANX (*n* = 249) and those without any diagnosis of ANX (*n* = 532), as well as *n* = 906 healthy controls (HC) from structural and diffusion-weighted MRI. The network-based statistic (NBS) toolbox was employed to evaluate network-level differences in structural connectivity among the three groups. Transdiagnostic analyses were conducted to explore the dimensional relationship between anxiety and structural connectivity. NBS revealed decreased structural connectivity in MDD patients without comorbid ANX and increased structural connectivity in MDD patients with comorbid ANX relative to HC, with both effects found in spatially overlapping white matter connections. Transdiagnostic analyses suggested that increases in anxiety were associated with increased structural connectivity across all groups. Our finding that hyperconnectivity rather than hypoconnectivity characterizes the structural connectome of MDD patients with comorbid ANX challenges the applicability of the dysconnection syndrome hypothesis to MDD with comorbid ANX, warranting symptom-based investigations of brain changes in mental disorders.

## Introduction

For over a decade, major depressive disorder (MDD) and anxiety disorders (ANX) have ranked among the ten most debilitating diseases worldwide [1], with lifetime prevalences of 16–20% and 14–29%, respectively [2–5]. Importantly, these disorders also frequently co-occur: up to 75% of individuals with a primary lifetime diagnosis of MDD also report a history of ANX [6] and up to 81% of those with primary ANX also experience comorbid MDD [7–9]. While overlapping diagnostic criteria may inflate this comorbidity [10, 11], its clinical significance is undeniable given its substantial contribution to the overall burden of illness [12]. Individuals with comorbid MDD and ANX (MDD + ANX) exhibit an earlier disease onset, more severe depressive symptoms, higher levels of suicidal ideation, greater functional impairments, and lower remission rates [4, 6, 7, 13–15]. Understanding the biological, psychological, and social factors underlying MDD + ANX is therefore crucial to advance mental health care for this severely affected population. The present study aims to contribute to this objective by investigating brain structural alterations in individuals with MDD and MDD + ANX.

From a neurobiological perspective, mental disorders have long been conceptualized as dysconnection syndromes [16–18]. This conceptualization suggests that symptoms arise from aberrant information transmission within the network of brain regions and their connecting white matter fiber tracts, collectively referred to as the human connectome [19, 20]. Previous research supports this by showing significant alterations in the structural connectome across MDD, bipolar disorder, and schizophrenia [21–25]. For instance, studies from our group revealed that, in MDD, dysconnectivity in the structural connectome typically presents as subtle yet widespread hypoconnectivity, particularly in individuals with earlier onset and more severe depressive symptoms [23, 26]. These findings reinforce the conceptualization that MDD may manifest as a dysconnection syndrome [27].

While connectome alterations in MDD have been thoroughly investigated, corresponding examinations in MDD + ANX are notably less common [28, 29]. Previous studies on connectome alterations in MDD, including our own, usually did not distinguish between MDD and MDD + ANX. This is problematic, as meta-analyses show that including participants with MDD + ANX significantly increases variability within the MDD-white matter association [30]. Moreover, individuals with MDD + ANX exhibit clinically distinct profiles compared to those with MDD alone, including earlier age of onset, more severe depressive symptoms, and higher rates of hospitalization [12]. These clinical markers have been associated with white matter alterations in prior research [23, 26, 31], suggesting that the neurobiological substrate may differ meaningfully between MDD and MDD + ANX.

To provide more precise insights, it is therefore necessary to distinguish between MDD and MDD + ANX. However, studies that made this distinction yielded inconsistent findings. Some reported reduced connectivity in white matter tracts in MDD + ANX compared to MDD and healthy controls (HC; [32, 33]), while others did not [34, 35]. These discrepancies may be attributed to methodological limitations: First, these studies involved small sample sizes of around 100 participants. While such samples may adequately power analyses of specific a priori hypotheses within predefined regions, they are likely insufficient for detecting reliable brain-wide associations when examining numerous tracts across the whole brain [36, 37]. Second, these studies employed tract-based frameworks (e.g., TBSS) that focus on localized alterations, potentially overlooking broader network-level changes [38]. Network-based frameworks, such as Network-Based Statistics (NBS; [39]), offer a different approach by simultaneously modeling the entire structural connectome within a graph-theoretical framework, enabling identification of spatially distributed subnetworks that may be more sensitive to subtle, widespread alterations across multiple pathways.

To overcome these limitations, the present study uses network analysis to investigate structural connectome differences in a large sample of HCs and individuals with MDD and MDD + ANX. While previous white matter studies in MDD + ANX yielded mixed results, the predominant pattern suggests reduced connectivity compared to HCs [32, 33]. We therefore hypothesize to find structural hypoconnectivity in MDD and MDD + ANX relative to HCs. For differences between MDD and MDD + ANX, existing evidence provides limited guidance. Given the more pronounced structural hypoconnectivity found in individuals with earlier disease onset and more severe depressive symptoms [23, 26], and given that individuals with MDD + ANX exhibit precisely these clinical characteristics [12], we anticipate that, compared to individuals with MDD, individuals with MDD + ANX will exhibit more pronounced hypoconnectivity.

Extending this diagnosis-based perspective, additional transdiagnostic analyses are conducted to explore the relationship between structural connectivity and dimensional measures of anxiety across HC, MDD, and MDD + ANX. These analyses provide a more nuanced, symptom-specific understanding of anxiety-related structural connectome alterations, potentially identifying alterations across the anxiety spectrum. We anticipate that individuals with higher levels of anxiety will show more pronounced structural hypoconnectivity regardless of diagnosis.

## Materials and methods

### Study procedures

Data were collected as part of the Marburg-Münster Affective Disorders Cohort Study (MACS, see [40] for a study protocol and [41] for an MRI quality assurance protocol). The study procedures received approval from the ethics committees at the Universities of Marburg and Münster, Germany. HCs, as well as in- and outpatients aged 18–65 years were recruited through newspaper advertisements or local psychiatric hospitals. All participants provided written informed consent and received financial compensation. Exclusion criteria are detailed in Supplementary 1.

### Clinical assessments

The Structured Clinical Interview for DSM-IV-TR Axis I (SCID-I; [42]) was administered to establish lifetime diagnoses of mental disorders. Participants were categorized into three groups: HC were included if they had no history of mental disorders. Individuals with MDD were included if they met criteria for a lifetime diagnosis of MDD but had no comorbid diagnoses for ANX. Individuals with MDD + ANX were included if they met criteria for a lifetime diagnosis of MDD and at least one comorbid ANX. Remission status was assessed according to SCID-I criteria.

Disease course information was collected through a semi-structured interview, including age of onset of first psychiatric symptoms, number of depressive episodes, and number of psychiatric hospitalizations.

### Self-report measures

Self-reported anxiety was assessed using the State-Trait Anxiety Inventory (STAI; [43]), a questionnaire comprising two 20-item scales measuring state anxiety and trait anxiety. State and trait sum scores were calculated, with higher scores indicating greater anxiety levels.

Current depressive symptoms were assessed using the Beck Depression Inventory-I (BDI-I; [44]) sum score for descriptive purposes.

### Acquisition and processing of MRI data

Two MR scanners were used for MRI data acquisition (see Supplement 2 for acquisition parameters and Supplementary 3 for preprocessing of diffusion-weighted images). We employed the CATO toolbox [45] to reconstruct the anatomical connectome by obtaining the nodes (114 cortical brain regions, depicted by the Cammoun subdivision of Freesurfer’s Desikan-Killiany atlas [46, 47]) from T1-weighted MRI and reconstructing the edges from diffusion-weighted MRI, defined as the number of reconstructed white matter streamlines between two nodes. See Supplementary 4 and 5 for details on the reconstruction and quality control procedures, respectively.

### Statistical analysis

Data analysis was conducted using Python 3.7.9 [48] and Matlab 2019b [49]. Figures were created using BrainNet Viewer [50], Simple Brain Plot [51], and Raincloud plots [52]. Statistical tests were conducted at a two-sided significance level of *α* = 0.05.

#### Structural connectome differences among diagnostic groups

To assess between-group differences in structural connectivity, we employed network-based statistic (NBS; [39]) analyses. NBS estimated a family-wise error (FWE)-corrected analysis of covariance to test for between-group differences (HC vs. MDD vs. MDD + ANX) in edge-wise connectivity (i.e., number of white matter streamlines) while correcting for age, sex, and scanner site. A primary NBS threshold of *F* = 4.0 was chosen in these analyses (see Supplementary 6 for details on NBS thresholds) and permutation tests (5000 permutations) were used to evaluate the significance of an identified network. In case of a significant effect, post-hoc *t*-tests were conducted to explore the pattern of between-group differences. In these post-hoc *t*-tests, we adjusted the significance level based on the false discovery rate (FDR) to account for multiple comparisons [53].

The topology of networks identified with NBS was characterized using two approaches. First, we identified network nodes with the highest number of affected edges. Second, we quantified the proportion of edges within the identified network connecting frontal, temporal, parietal, occipital, and insular brain regions and compared this to the respective proportions of edges in the entire connectome (derived by counting all edges present in at least 5% of the sample). Third, to assess whether identified networks involved predominantly short-range or long-range connections, we computed the average white matter fiber length within each identified network. This was calculated as the mean total fiber length across participants, where each participant’s total fiber length represented the sum of individual fiber lengths across all edges within the identified network. Using a permutation test, we compared this observed average total fiber length against the distribution of average total fiber lengths from 1000 topology-preserving random networks of identical size.

To verify that observed group differences were not driven by methodological artifacts, we conducted several robustness checks. We evaluated consistency across (1) sexes by repeating analyses separately for males and females; (2) study sites by conducting site-specific analyses; and (3) remission status by separately analyzing acutely depressed individuals and those with partially or fully remitted episodes. We further assessed potential confounds by (4) controlling for non-linear age effects (age²); (5) including head motion parameters (from FSL’s eddy output) as covariates; and (6) excluding connectome outliers identified using established quality control procedures for structural connectivity matrices (see Supplementary 5) [54]. Finally, we verified findings using (7) an alternative connectivity measure (streamline volume density corrected for gray matter volumes) and (8) the original Desikan-Killiany atlas including subcortical regions.

#### Brain-wide distribution of structural connectivity differences

To examine structural connectivity differences between HC, MDD, and MDD + ANX beyond the identified subnetworks, we calculated age-, sex-, and site-corrected *t*values for nodal estimates of structural connectivity (i.e., the mean number of streamlines connected to a given brain region). We computed *t*-values for the contrasts between HC and MDD and between HC and MDD + ANX. The resulting *t*-values were then mapped onto the brain surface for visualization.

Additionally, we conducted meta-analyses of these *t*-values using two approaches: First, we evaluated the proportion of brain regions showing hypoconnectivity (*t* < 0) versus hyperconnectivity (*t* > 0) for each contrast using chi-square tests. Second, we compared the *t*-values from the HC vs. MDD contrast to those from the HC vs. MDD + ANX contrast using a paired *t*-test.

#### Transdiagnostic associations between structural connectivity and anxiety

Continuous associations between structural connectivity and anxiety were tested exploratorily using NBS. Here, NBS estimated two FWE-corrected generalized linear models to test the association between structural connectivity and either state or trait anxiety while correcting for age, sex, and scanner site across HC, and individuals with MDD and MDD + ANX. Permutation tests were used to evaluate network significance, and *p*-values were adjusted for multiple comparisons based on the FDR. If either of the two undirected generalized linear models identified a network of white matter tracts associated with either state or trait anxiety, a corresponding directed linear regression model was estimated in NBS to explore the primary direction of that association.

## Results

### Demographic and clinical characteristics of the sample

Our sample consisted of *n* = 906 HC, *n* = 532 individuals with MDD, and *n* = 249 individuals with MDD + ANX (see Table 1). Comorbid anxiety diagnoses (according to DSM-IV) included agoraphobia (*n* = 7), generalized anxiety disorder (*n* = 31), obsessive-compulsive disorder (*n* = 23), panic disorder (*n* = 65), posttraumatic stress disorder (*n* = 62), social anxiety disorder (*n* = 90), and specific phobias (*n* = 56), with *n* = 112 (45%) individuals being diagnosed with more than one anxiety disorder.

**Table 1:** Demographic and clinical characteristics of the study sample.

	HC (*n* = 906)	MDD (*n* = 532)	MDD + ANX (*n* = 249)	Statistic	*p*value
Age	34.10 (12.81)	37.54 (13.34)	34.55 (12.14)	*F* = 12.40	<0.001
Sex (female : male)	576:330 64:36%	319:213 60:40%	182:67 73:27%	χ^*2*^=12.73	0.002
Remission status (acute : partial : full remission)		224:129:179 42:24:34%	122:69:5849:28:23%	*F* = 8.61	0.014
BDI-I	4.08 (4.30)	15.82 (10.80)	20.94 (10.64)	*F* = 623.16	<0.001
STAI-S	34.40 (8.36)	48.50 (12.85)	53.70 (11.63)	*F* = 496.38	<0.001
STAI-T	33.61 (8.34)	51.23 (12.50)	57.36 (10.58)	*F* = 809.63	<0.001
Age of onset		27.93 (12.77)	21.64 (10.72)	*t* = 7.16	<0.001
Number of hospitalizations		1.43 (1.98)	1.80 (2.04)	*t* = -2.35	0.019
Number of depressive episodes		3.37 (4.12)	4.95 (8.99)	*t* = -2.57	0.011

Demographic and clinical characteristics of healthy controls (HC) and depressed individuals without (MDD) and with comorbid anxiety disorder (MDD + ANX). BDI-I=Beck Depression Inventory; STAI-S and STAI-T=State and trait subscales from the State-Trait Anxiety Inventory.

Diagnostic groups differed in mean age, sex, and site distribution (see Table 1). Individuals with MDD + ANX were predominantly female and reported an earlier disease onset, a higher number of depressive episodes, and more severe depressive symptoms (according to the BDI-I) compared to those with MDD. The latter was also reflected in a higher proportion of acutely depressed individuals in the MDD + ANX group and a higher proportion of individuals in full remission in the MDD group. Details on patient medications and comorbid disorders are provided in Supplementary 7 and 8.

### Network-based differences in structural connectivity among diagnostic groups

Our NBS analysis identified significant components of the structural connectome capturing differences in edge-wise structural connectivity between HC, MDD, and MDD + ANX (NBS *F*-threshold *F* = 4.0, p_FWE_ = 0.008, partial *η²*=0.009). Post-hoc *t*-tests revealed that these differences were driven by significant decreases in structural connectivity in individuals with MDD (NBS *t*-threshold *t* = –1.962, p_FWE,FDR_ < 0.05, 42 edges, Cohen’s *d* = –0.467) and significant increases in structural connectivity in individuals with MDD + ANX (NBS *t*-threshold *t* = 1.962, *p*_*FWE,FDR*_ < 0.05, 170 edges, Cohen’s *d* = 0.759) relative to HC. Consequently, we also identified a subnetwork characterized by significant increases in structural connectivity in individuals with MDD + ANX as compared to those with MDD, though at a lower threshold (NBS *t*-threshold *t* = 1.9, *p*_*FWE,FDR*_ < 0.05, 126 edges, Cohen’s *d* = 0.735).

**Fig. 1:**
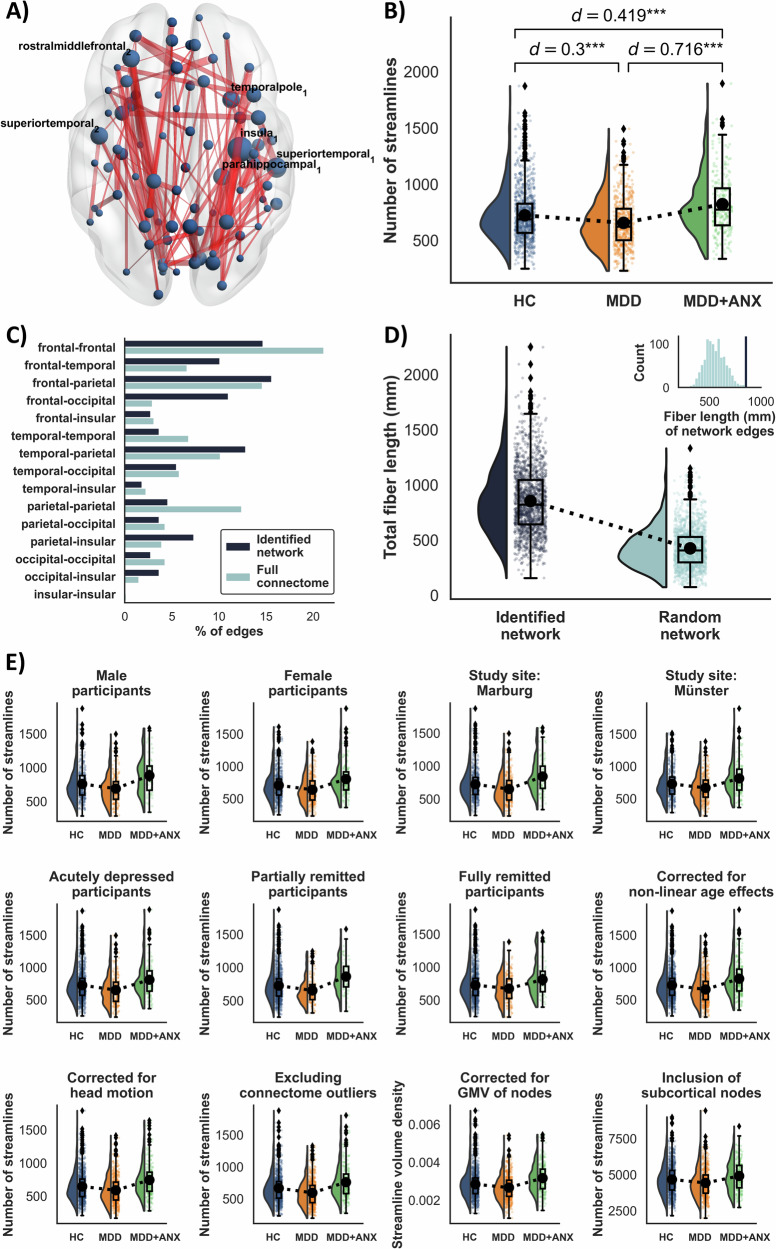
Subnetwork of the structural connectome with decreased connectivity in depressed individuals without anxiety disorder (MDD) and increased connectivity in depressed individuals with comorbid anxiety disorder (MDD + ANX), as compared to healthy controls (HC). Network of white matter fiber tracts capturing differences among healthy controls (HC) as well as depressed individuals without (MDD) and with comorbid anxiety disorder (MDD + ANX). **A** Axial view of the network identified via NBS. Node size represents the number of affected edges connected to each region, with larger nodes indicating hub regions within the network. Nodes with the highest number of affected edges are labeled with their anatomical region names. Edge thickness reflects the effect size (t statistic) for each connection, with thicker edges indicating stronger group differences. **B** Between-group differences in the identified network, with effect sizes shown as Cohen’s d. **C** Frequency of edges connecting frontal, temporal, parietal, occipital, and insular regions in the identified network, compared to the frequency of the respective edges in the full connectome. **D** Individual estimates of total fiber length across all edges in the identified network versus the first of 1000 size- and topology-matched random networks, with comparison of mean total fiber length (averaged across individuals) in the identified network to mean total fiber lengths in all 1000 randomly selected networks (upper right corner). **E** Pattern of between-group differences in robustness checks (see Supplementary 9 for effect sizes). Robustness checks included: (1) separate analyses for males and females; (2) separate analyses for participants from either study site; (3) separate analyses for participants with acute, partially remitted or remitted depressive episode; (4) correcting for non-linear age effects; (5) including head motion as a covariate (derived from FSL’s eddy output); (6) excluding connectome outliers; (7) using an alternative measure of structural connectivity (streamline volume density) corrected for gray matter volumes; and (8) using the original Desikan-Killiany atlas that includes subcortical regions. See online version for colored figures.

To explore whether these decreases and increases in structural connectivity were detectable within an interconnected subnetwork of edges, we performed an additional, directed NBS analysis specifically testing for the observed pattern of MDD < HC < MDD + ANX. This analysis identified a subnetwork that precisely matched this pattern (NBS *t*-threshold *t* = 1.962, *p*_*FWE*_ = 0.028, 109 edges, see Fig. 1A, B), meaning that in each edge of that network, individuals with MDD exhibited decreased connectivity and individuals with MDD + ANX exhibited increased connectivity compared to HC. Nodes with the highest number of affected edges of that network were localized within the right insula, right superior temporal gyrus, left rostral middle-frontal gyrus, left superior temporal gyrus, right parahippocampal gyrus, and right temporal pole. The network mainly comprised fronto-frontal, fronto-parietal, and temporo-parietal edges (14.7%, 15.6%, and 12.8% of network edges, respectively). However, comparisons of the identified network with the distribution of edges present in the entire connectome also revealed a disproportionately high number of affected edges connecting frontal and occipital brain regions (11%; $\chi$^2^ = 43.70, *p* < 0.001, see Fig. 1C), suggesting a particular involvement of long-range connections. This was corroborated by a significantly higher average white matter fiber length within the identified network compared to the average white matter fiber lengths from 1000 topology-preserving random networks of identical size (*p*_perm_ = 0.006, see Fig. 1D).

In robustness checks, we verified that the observed pattern of increased and decreased connectivity in individuals with MDD and MDD + ANX was not driven by (1) sex-specific differences in structural connectivity; (2) differences between study sites; (3) the participants’ remission status; (4) non-linear age effects; (5) the participants’ head movements during MRI acquisition; (6) outliers in structural connectivity; (7) differences in gray matter volume of the connected brain regions; or (8) our decision of excluding subcortical brain regions from our analysis; None of these approaches changed the pattern of results (see Fig. 1E), with effect sizes across robustness checks ranging between Cohen’s *d* = −0.33 to –0.23 (HC vs. MDD), *d* = 0.23 to 0.60 (HC vs. MDD + ANX), and *d* = 0.46 to 0.83 (MDD vs. MDD + ANX; see Supplementary 9). Furthermore, we examined the extent to which the choice of the primary NBS threshold influenced our results (see Supplementary 10). As expected, less stringent thresholds yielded more widespread networks; however, the overall pattern of findings remained consistent, with effect sizes ranging between Cohen’s *d* = −0.30 to −0.41 (HC vs. MDD), *d* = 0.27 to 0.42 (HC vs. MDD + ANX), and *d* = 0.65 to 0.72 (MDD vs. MDD + ANX).

### Brain-wide distribution of structural connectivity differences

**Fig. 2:**
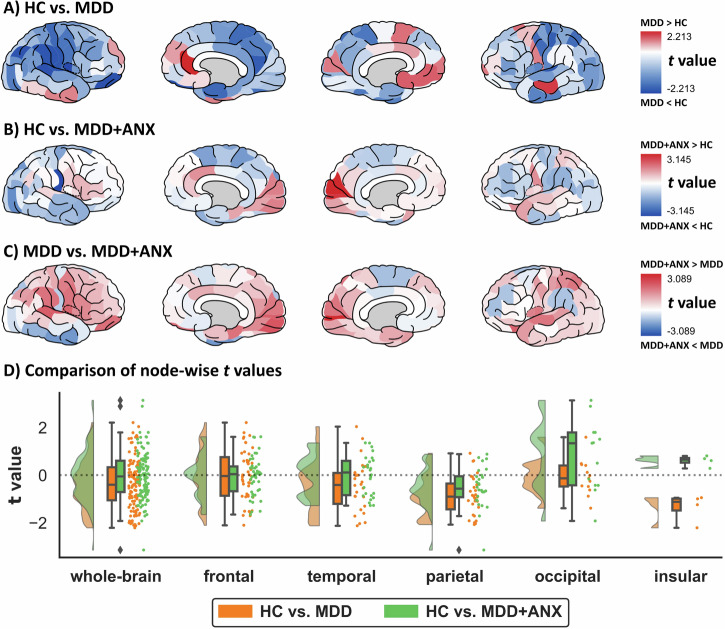
Brain-wide distributions of differences in node-wise structural connectivity between healthy controls (HC) and depressed individuals without (MDD) and with comorbid anxiety disorders (MDD + ANX). Spatial distribution of between-group differences (t-values) in nodal structural connectivity (mean number of streamlines connected to a given node). **A** Differences between healthy controls (HC) and individuals with depression but without comorbid anxiety disorder (MDD). **B** Differences between HC and individuals with depression and comorbid anxiety disorder (MDD + ANX). **C** Differences between MDD and MDD + ANX. **D** Comparison of t-values for nodal structural connectivity differences between HC and MDD to those between HC and MDD + ANX across all 114 nodes (“whole-brain”), and separately within frontal, temporal, parietal, occipital, and insular brain regions. See online version for colored figures.

We examined the spatial distribution of structural connectome alterations across the entire brain by analyzing nodal estimates of structural connectivity (see Fig. 2A–C). Descriptively, structural hypoconnectivity (i.e., reductions in connectivity relative to HC) was less prevalent in the structural connectome of individuals with MDD + ANX (53% of brain regions) than in those with MDD (67% of brain regions). Conversely, increases in connectivity were found more widespread across the structural connectome of individuals with MDD + ANX (47% of brain regions) than in those with MDD (33% of brain regions, $\chi$^2^ = 4.67, *p* = .031). These differences resulted in a substantial proportion of the structural connectome of individuals with MDD + ANX showing increased structural connectivity compared to individuals with MDD (59% of brain regions). Meta-analytical comparisons revealed that node-wise *t*-values were indeed significantly lower in the HC vs. MDD contrast as compared to the HC vs. MDD + ANX contrast (paired *t*test *t* = –2.942, *p* = 0.002, Cohen’s *d* = 0.333), with particular differences in the connectivity of occipital and insular brain regions (see Fig. 2D).

### Transdiagnostic associations between structural connectivity and anxiety

**Fig. 3:**
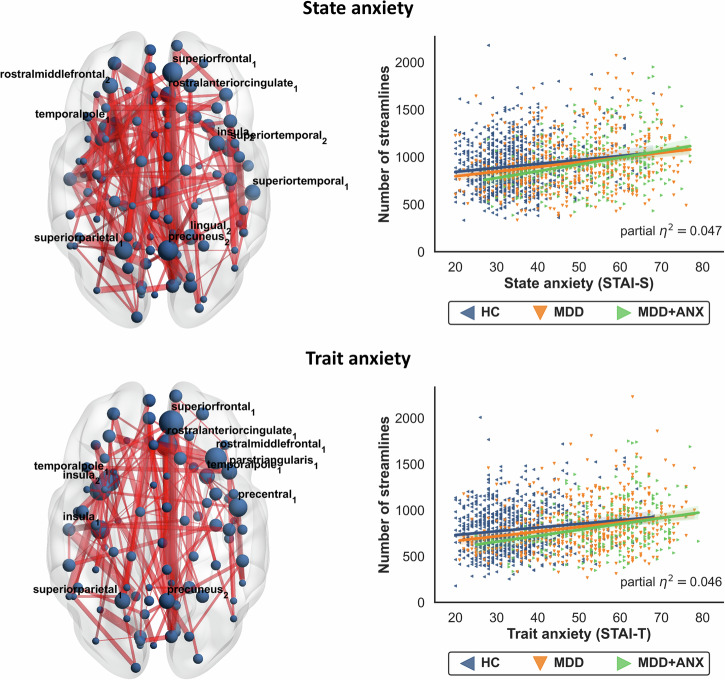
Subnetworks of the structural connectome associated with state and trait anxiety across healthy controls (HC) and depressed individuals without (MDD) and with comorbid anxiety disorders (MDD + ANX). Node size represents the number of affected edges connected to each region (degree), with larger nodes indicating hub regions within the network. Nodes with the highest number of affected edges are labeled with their anatomical region names. Edge thickness reflects the effect size (F-statistic) for each connection, with thicker edges indicating stronger group differences. See online version for colored figures.

Our transdiagnostic analyses identified subnetworks of edges where structural connectivity was significantly associated with state and trait anxiety across all participants (state anxiety: NBS *F*-threshold *F* = 4.0, *p*_*FWE,FDR*_ < 0.05, 186 edges; trait anxiety: NBS *F*-threshold *F* = 4.0, *p*_*FWE,FDR*_ < 0.05, 162 edges). Post-hoc directed linear regression models revealed that these associations predominantly followed a positive direction (state anxiety: NBS *t*-threshold *t* = 1.962, *p*_*FWE*_ < 0.05, 154 edges, partial *η*^*2*^ = 0.005; trait anxiety: NBS *t*-threshold *t* = 1.962, *p*_*FWE*_ < 0.05, 118 edges, partial *η*^*2*^ = 0.005; see Fig. 3), indicating that higher levels of anxiety were associated with increases in structural connectivity across HC, MDD, and MDD + ANX.

## Discussion

Individuals with MDD + ANX experience earlier disease onset, more severe depressive symptoms, and greater functional impairments than those with MDD alone. Despite this significant clinical burden, the neurobiological underpinnings of MDD + ANX remain poorly understood. The present study investigated structural connectome alterations in a large sample of HC and individuals diagnosed with MDD or MDD + ANX. Contrary to our hypotheses, our analyses revealed that the structural connectome in individuals with MDD + ANX is at least partially defined by *hyper*connectivity rather than *hypo*connectivity. Transdiagnostic analyses indicated that this pattern even extends to HCs and individuals with MDD with elevated levels of anxiety but without ANX. In the following, we will discuss these findings and explore broader implications for future network neuroscience research in mental health.

Previous research on connectome alterations in mental disorders has identified marked patterns of structural hypoconnectivity, i.e., reduced connectivity, particularly in those with an earlier age of onset and more severe depressive symptoms [21, 23, 26]. In the present study, individuals with MDD indeed exhibited this expected pattern of hypoconnectivity. However, those with MDD + ANX displayed a contrasting pattern of structural hyperconnectivity, which was unexpected given their earlier onset and higher depressive symptom severity. To ensure the robustness of this finding, we conducted several analyses to rule out potential confounding factors, such as sex differences or methodological artifacts. Across all these analyses, the hyperconnectivity in individuals with MDD + ANX persisted. Importantly, this pattern was consistently found across different analytical approaches, such as subnetwork-specific and brain-wide analyses, and across observer-rated diagnoses as well as self-reported anxiety data. Post hoc analyses further revealed that these alterations converged on long-range connections and particularly affected insular brain regions. The robustness and spatial consistency of these results suggest that they are not merely artifacts of specific methods or assessments.

Our finding of hyperconnectivity associated with elevated trait and state anxiety raises the question of whether these results reflect stable trait characteristics or rather transient state effects. While our diagnosis-based analysis might suggest a trait effect, there is evidence that sustained elevations in current affective symptoms can indeed be linked with alterations in structural connectivity [26]. However, in the present study, a definitive examination of state versus trait effects of anxiety was limited by the high intercorrelation between these measures, which precludes clear separation of their unique contributions. Moreover, cross-sectional DWI studies are inherently constrained in their ability to distinguish between state and trait effects [55]. To determine whether the observed hyperconnectivity is attributable primarily to trait factors that precede disease onset or to (potentially sustained) state effects emerging during illness progression, longitudinal designs with repeated assessments are required [55].

The mechanisms underlying the identified pattern of structural hyperconnectivity remain speculative, given the lack of large-scale studies on structural connectivity alterations in MDD + ANX. Hypotheses on these mechanisms may be drawn from other modalities, such as functional connectivity. One possibility involves a compensatory mechanism, wherein hyperconnectivity in certain connections offsets hypoconnectivity elsewhere in the connectome [38, 56]. However, the higher disease severity in individuals with MDD + ANX questions the effectiveness of this compensation. An alternative explanation may lie in hyperconnectivity resulting from anxiety-related hypervigilance and heightened threat response: For instance, previous studies have linked increased functional connectivity to early life adversity in rodents [57] and heightened threat response in generalized anxiety disorder [58], as well as hyperactivation of the fear circuit in social anxiety disorder [59]. Given the known relationship between functional and structural connectivity [60, 61], functional hyperconnectivity may, over time, translate into structural hyperconnectivity through Hebbian plasticity [62]. Future large-scale studies could examine whether functional hyperconnectivity following dysfunctional threat response precedes structural hyperconnectivity in MDD + ANX.

While our findings suggest that the nature of dysconnectivity in MDD—whether reduced or increased—depends on the individual clinical profile, we do not propose that these patterns could serve as reliable biomarkers for differential diagnosis. Consistent with prior studies, the effect sizes observed in our study are small, rendering our findings insufficient for differentiating between diagnoses at the individual level [23, 63, 64]. This limited effect size may reflect individual deviations from group-level patterns, for instance, due to comorbidity with other mental disorders, heterogeneity in depressive symptom profiles, and the individually diverse locations of neurobiological alterations in mental disorders [65–68]. Given this variability, reliably detecting structural connectome differences across all combinations of diagnoses and comorbidities would require enormous sample sizes, rendering traditional group comparisons impractical.

To advance psychiatric network neuroscience, future studies should focus on identifying transdiagnostic biomarkers for specific symptoms rather than relying on diagnostic categories [69], embedded in frameworks such as the Research Domain Criteria [70, 71]. Our transdiagnostic analysis of anxiety-related connectome alterations exemplifies this approach. In future research, this strategy should be expanded to address multiple symptom dimensions simultaneously, thereby identifying alterations that are truly symptom-*specific*. Integrating individual symptom networks, which model the individual co-occurrence of psychiatric symptoms [72], with brain networks in multi-layer networks may offer a promising avenue for this line of research [73]. Such approaches may help determine if structural hyperconnectivity is linked to specific symptom clusters or biological profiles, such as immuno-metabolic depression [74]. Normative modeling may further enhance these strategies by identifying brain structural alterations relative to normative variations at the subject-level [75]. This combination of methods may help unravel the neurobiological basis of specific symptoms, ultimately paving the way for symptom-tailored interventions rooted in neurobiology [76, 77].

Several limitations should be noted. First, our analyses focused on anxiety as a broad syndrome without further differentiation. Future research should investigate whether different symptoms exhibit distinct structural connectome signatures. Second, our study lacks a group of patients with ANX but without MDD. Based on our findings, we would expect an even more pronounced pattern of structural hyperconnectivity in this group. Third, field maps for distortion correction were not available for this dataset, which may have affected the precision of our tractography results, particularly in regions prone to susceptibility-induced distortions such as orbitofrontal and anterior temporal areas. Fourth, the relatively smaller sample size of the MDD + ANX group may raise concerns. However, it is unlikely to account for the observed pattern of hyperconnectivity, as increases in structural connectivity were also observed in our transdiagnostic analysis covering the entire sample. Finally, this study was not pre-registered, and findings should therefore be interpreted as exploratory and require replication in independent samples.

In conclusion, our findings shed light on the nature of structural connectome alterations in MDD and MDD + ANX, challenging the traditional narrative of dysconnectivity as a linear marker of depressive symptom severity. The unexpected discovery of hyperconnectivity in individuals with MDD + ANX, a group with more severe depressive symptoms, underscores the need for a paradigm shift in psychiatric network neuroscience that prioritizes individualized, symptom-specific research.

## Supplementary information

Supplementary Material

## Data Availability

All data are available in the main text or the supplementary materials. Participant data used in this study, informed consent forms, and scripts employed for analysis will be made available upon reasonable request to the corresponding author.
