# Supplementary material for: Network-based analysis of differential white matter connectivity in major depressive disorder with and without comorbid anxiety
Source: Neuropsychopharmacology. 2026 Jan 12;51(5):916–25. doi: 10.1038/s41386-025-02312-y (PMC13013687; doi:10.1038/s41386-025-02312-y)
Supplement: Supplementary file 1 — Supplementary Material [file 41386_2025_2312_MOESM1_ESM.docx]

Supplement to: Network-Based Analysis of Differential White Matter Connectivity in Major Depressive Disorder with and without Comorbid Anxiety

[Supplement 1: Exclusion criteria 2](#_Toc216104064)

[Supplement 2: MRI acquisition parameters 2](#_Toc216104065)

[Supplement 3: Preprocessing of diffusion-weighted images 2](#_Toc216104066)

[Supplement 4: Connectome reconstruction procedure 3](#_Toc216104067)

[Supplement 5: Quality control procedure for connectivity matrices 4](#_Toc216104068)

[Supplement 6: Information on the primary NBS threshold 5](#_Toc216104069)

[Supplement 7: Patients’ medication intake 8](#_Toc216104070)

[Supplement 8: Comorbid diagnoses among patients 9](#_Toc216104071)

[Supplement 9: Effect sizes for between-group differences in robustness checks 10](#_Toc216104072)

[Supplement 10: Networks identified at different NBS thresholds 10](#_Toc216104073)

[Supplemental References 13](#_Toc216104074)

# Supplement 1: Exclusion criteria

Exclusion criteria comprised the presence of any neurological abnormalities, history of seizures, head trauma or unconsciousness, severe physical impairment (e.g., cancer, unstable diabetes, epilepsy, etc.), pregnancy, hypothyroidism without adequate medication, claustrophobia, color blindness, and general MRI contraindications (e.g., metallic objects in the body). Further, lifetime diagnoses of alcohol dependence posed a reason for exclusion.

# Supplement 2: MRI acquisition parameters

Both T1 and DTI data were acquired using a 3T whole body MRI scanner (Marburg: Tim Trio, 12-channel head matrix Rx-coil, Siemens, Erlangen, Germany; Münster: Prisma, 20-channel head matrix Rx-coil, Siemens, Erlangen, Germany). A GRAPPA acceleration factor of two was employed for both sequences. A high-resolution T1-weighted dataset was acquired using a 3D-MPRAGE-sequence (TE_Münster_=2.28 ms, TE_Marburg_=2.26 ms, TR_Münster_=1900 ms, TR_Marburg_=2130 ms TI=900 ms) with an isotropic voxel size of 1x1x1 mm³. For DTI imaging, fifty-six axial slices with no gap were measured with an isotropic voxel size of 2.5x2.5x2.5 mm³ (TE=90 ms, TR=7300 ms). Five non-DW images (b=0 s/mm²) and 2 x 30 DW images with a b-value of 1000 s/mm² were acquired. Imaging pulse sequence parameters were standardized across both sites to the extent permitted by each platform.

# Supplement 3: Preprocessing of diffusion-weighted images

Diffusion-weighted images (DWI) were realigned and corrected for eddy currents (Andersson & Skare, 2002) using the eddy function from FSL 6.0.1 [(Andersson & Sotiropoulos, 2016; Woolrich et al., 2009)](https://www.zotero.org/google-docs/?JxuoVC). Diffusion tensor imaging models the measured signal of a voxel using a single tensor, which describes the diffusion signal as having a preferred diffusion direction per voxel. The CATO toolbox [(de Lange et al., 2023)](https://www.zotero.org/google-docs/?AKVALf) employed for the reconstruction of the anatomical connectome (see Supplement 4) uses the informed RESTORE algorithm [(Chang et al., 2005, 2012)](https://www.zotero.org/google-docs/?Q0dsuO) that estimates the tensor while identifying and removing outliers during the fitting, thereby reducing the impact of physiological noise artifacts on the DTI modeling. Based on the diffusion profiles, white matter pathways were reconstructed using deterministic tractography. To this end, eight seeds were started per voxel, and for each seed, a tractography streamline was constructed by following the main diffusion direction from voxel to voxel. Stop criteria included reaching a voxel with a fractional anisotropy <0.1, making a sharp turn of >45°, reaching a gray matter voxel, or exiting the brain mask [(Van Den Heuvel et al., 2013)](https://www.zotero.org/google-docs/?O6UYBj).

# Supplement 4: Connectome reconstruction procedure

We employed the publicly available CATO toolbox [(de Lange et al., 2023)](https://www.zotero.org/google-docs/?30mDam) for reconstructing the anatomical connectome. The procedure included the following steps:

We obtained a network of 114 brain regions along with the reconstructed white matter streamlines between these brain regions for each participant. To identify the brain regions, we relied on FreeSurfer's Desikan-Killiany atlas [(Cammoun et al., 2012; Desikan et al., 2006; Hagmann et al., 2008)](https://www.zotero.org/google-docs/?7RhZh6). However, given the poorer DWI signal-to-noise ratio in subcortical regions and the dominant effect of these regions on network properties, we decided to use a subdivision of this atlas containing only cortical regions, as we have done in previous work [(Gruber et al., 2023; Repple et al., 2020, 2023)](https://www.zotero.org/google-docs/?Xa7Cau).

To reconstruct the streamlines, we applied a deterministic streamline tractography based on the Fiber Assignment by Continuous Tracking (FACT) algorithm [(Mori & van Zijl, 2002)](https://www.zotero.org/google-docs/?CsyTbM). We chose this deterministic algorithm instead of more advanced diffusion direction reconstruction methods because it provides a reasonable balance between false-negative and false-positive fiber reconstructions [(Sarwar et al., 2019)](https://www.zotero.org/google-docs/?fjPieb). Connections between two nodes, i.e., brain regions, were included if at least three reconstructed streamlines connected them. This type of thresholding was applied since we wanted to balance the sensitivity and specificity of the resulting connectivity matrices [(de Reus & van den Heuvel, 2013; Zalesky et al., 2016)](https://www.zotero.org/google-docs/?2Ehasa).

Each participant's network was finally stored in a connectivity matrix with rows and columns representing nodes (i.e., brain regions) and matrix entries representing edges (i.e., connectivity strength measured as the number of reconstructed streamlines) between two nodes.

# Supplement 5: Quality control procedure for connectivity matrices

To ensure the quality of the connectivity matrices, we followed the approach from [(Van Den Heuvel et al., 2019)](https://www.zotero.org/google-docs/?pk3UJ3) and applied several criteria to identify outliers within the matrices. The criteria for outlier detection included 1) the average number of streamlines, 2) the average fractional anisotropy, 3) the average prevalence of each participant's connections (low value if the participant has "odd" connections), and 4) the average prevalence of each participant's connected brain regions (high value if the participant misses frequent connections). For each metric, quartiles (Q1, Q2, Q3) and interquartile range (IQR=Q3-Q1) were calculated. A data point was declared an outlier if its value was below Q1-1.5*IQR or above Q3+1.5*IQR for any of the four metrics.

We adopted a conservative approach by retaining all identified outliers (*n*=52) in the primary analyses, as distinguishing artifactual data from meaningful biological variation is challenging. Routine exclusion of flagged cases may inappropriately discard valid data. The impact of outliers was instead evaluated in a dedicated robustness analysis through their exclusion. Results remained consistent regardless of outlier inclusion (see Results), indicating that atypical connectivity matrices did not drive our findings. Additionally, the NBS permutation testing framework is inherently robust to outliers, as significance is assessed relative to the empirical sample distribution.

# Supplement 6: Information on the primary NBS threshold

In the present network-based statistic (NBS) analyses, we used a primary threshold of *F*=4.0 and *t*=1.96, ensuring that each individual edge was associated with the effect of interest at *p*<0.05. The primary threshold in NBS determines the initial test statistic cut-off for identifying connections within the brain network associated with the effect of interest. However, it is important to note that it does not directly influence the statistical significance of the identified network [(Zalesky et al., 2010)](https://www.zotero.org/google-docs/?aocVyk). The significance of the network is ultimately determined through the permutation test, which controls for the family-wise error rate (FWER) across different thresholds.

The threshold primarily serves to describe the nature of the identified effect [(*Reference Manual for NBS Connectome (v1.2)*, 2012)](https://www.zotero.org/google-docs/?euKaa6). Lower thresholds (e.g., thresholds corresponding to *p*<0.05) tend to reveal widespread but subtle effects, capturing broader network alterations with weaker, diffuse associations. In contrast, higher thresholds (e.g., thresholds corresponding to *p*<0.001) focus on stronger, more localized effects, revealing more focal network changes. Effects that emerge across a range of lower and higher thresholds may reflect a combination of both subtle, widespread, and stronger, focal alterations.

To illustrate the stability of the identified networks, we provide line plots depicting the relationship between the chosen threshold and network size (i.e., the number of edges). These plots demonstrate that the networks remain stable across consecutive thresholds, further supporting the robustness of our findings and emphasizing that the chosen threshold primarily affects the sensitivity of the method rather than the statistical significance of the identified network itself. Consequently, whether a given effect is detected does not depend on the precise threshold selected; rather, the fact that effects are identified across a range of relatively low thresholds indicates that the observed alterations in structural connectivity represent relatively subtle but widespread connectome alterations.

| **Figure S1.** Relationship between the primary threshold applied in NBS analyses on group differences and the size of the identified network. |
| --- |
| 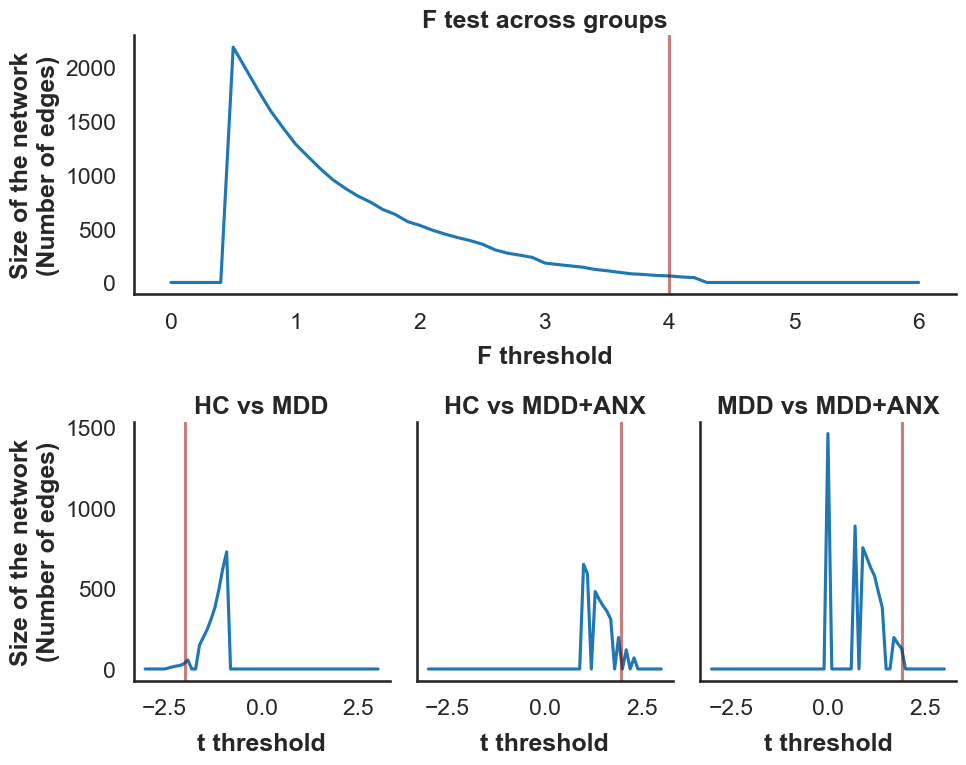 |
| *Note.* Panel 1 (top row) shows the association between threshold and network size for a covariate analysis performed in NBS, comparing differences in structural connectivity between healthy individuals (HC) and depressed individuals without (MDD) and with comorbid anxiety disorder (MDD+ANX) while controlling for age, sex, and study site. Panels 2–4 (bottom row) display the same association for post-hoc *t*-tests in NBS, showing pairwise comparisons between the three groups. The red lines indicate the thresholds used in the primary analysis, as described in the article. |

| **Figure S2.** Relationship between primary threshold applied in NBS analyses on transdiagnostic structural connectivity associations with state (STAI-S) or trait (STAI-T) anxiety and the size of the identified network. |
| --- |
| 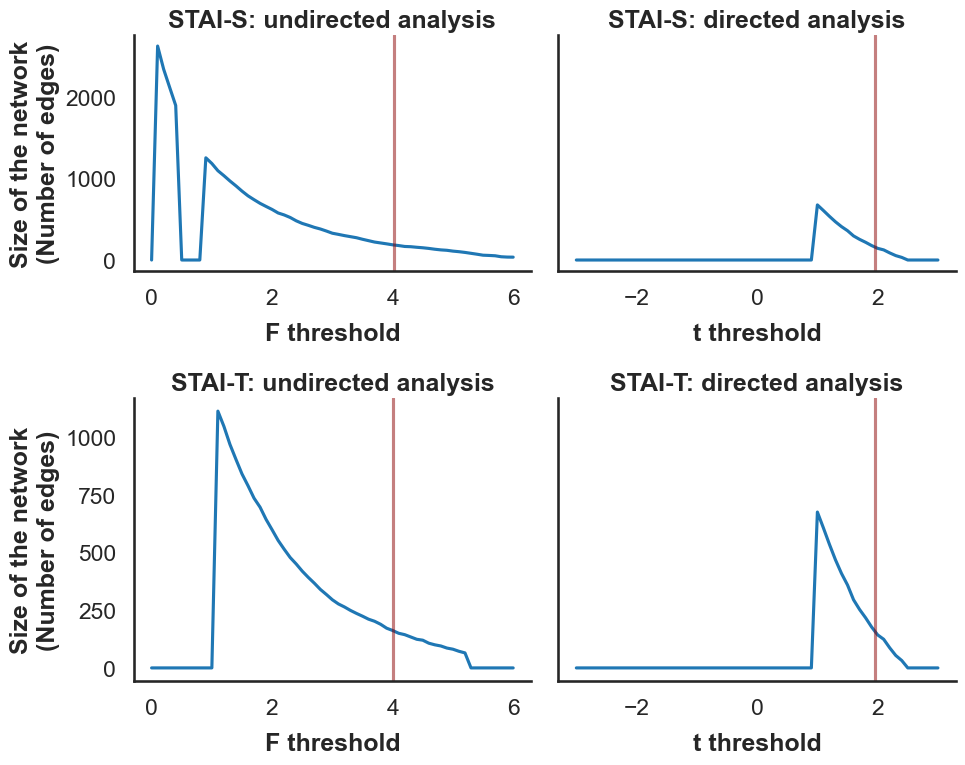 |
| *Note.* Panel 1 (top row) shows the association between threshold and network size for an undirected regression analysis conducted in NBS, examining the relationship between structural connectivity and state anxiety (STAI-S). Panel 2 (top row) presents the association for a directed regression analysis (i.e., yielding either positive or negative associations), exploring the same relationship between structural connectivity and state anxiety (STAI-S). The plots in the second row display the corresponding associations for trait anxiety (STAI-T), with Panel 3 showing the results of an undirected regression analysis and Panel 4 presenting the results of a directed regression analysis. All four analyses were conducted across all participants and corrected for age, sex, and scanner-site. The red lines indicate the thresholds used in the primary analysis, as described in the article. |

# Supplement 7: Patients’ medication intake

| **Table S1.** Usage of psychopharmacological drugs among depressed individuals without (MDD) and with a comorbid anxiety disorder (MDD+ANX) in absolute (n) and relative (%) numbers. | | | | | |
| --- | --- | --- | --- | --- | --- |
| Medication | **MDD (*n*=532)** | |  | **MDD+ANX (*n*=249)** | |
|  | ***n*** | ***%*** |  | ***n*** | ***%*** |
| *Antidepressants* | | | | | |
| SNRI | 133 | 25 |  | 68 | 27 |
| SSRI | 126 | 24 |  | 72 | 29 |
| NDRI | 14 | 3 |  | 9 | 4 |
| NaSSA | 47 | 9 |  | 18 | 7 |
| NaRI | 1 | 0 |  | 0 | 0 |
| TZA | 22 | 4 |  | 15 | 6 |
| MAO | 3 | 1 |  | 0 | 0 |
| Agomelatine | 19 | 4 |  | 12 | 5 |
| Lithium | 12 | 2 |  | 4 | 2 |
| Antipsychotics | 99 | 19 |  | 52 | 21 |
|  | | | | | |

# Supplement 8: Comorbid diagnoses among patients

| **Table S2.** Comorbid diagnoses among depressed individuals without (MDD) and with comorbid anxiety disorder (MDD+ANX) | | | |
| --- | --- | --- | --- |
| **Diagnosis** | **Specification** | **MDD** | **MDD+ANX** |
| Anxiety disorder | Agoraphobia without history of panic disorder | 0 | 7 |
|  | Generalized anxiety disorder | 0 | 31 |
|  | Obsessive-compulsive disorder | 0 | 23 |
|  | Panic disorder - Without agoraphobia | 0 | 29 |
|  | Panic disorder - With agoraphobia | 0 | 36 |
|  | Posttraumatic stress disorder | 0 | 62 |
|  | Social phobia | 0 | 90 |
|  | Specific phobia | 0 | 56 |
| Depressive disorder | Dysthymic disorder | 15 | 18 |
| Eating disorder | Bulimia nervosa | 16 | 7 |
|  | Eating disorder not otherwise specified | 10 | 10 |
| Impulse-Control Disorder | Pathological Gambling | 0 | 1 |
| Schizophrenia and other psychotic disorder | Brief psychotic disorder | 3 | 1 |
|  | Delusional disorder | 2 | 0 |
|  | Psychotic disorder not otherwise specified | 0 | 1 |
| Somatoform disorder | Hypochondriasis/Illness anxiety disorder | 2 | 1 |
|  | Pain disorder - Associated with both psychological factors and a general medical condition | 5 | 0 |
|  | Pain disorder - Associated with psychological factors | 5 | 6 |
|  | Somatization disorder | 0 | 2 |
|  | Undifferentiated somatoform disorder | 3 | 2 |
| Substance-related disorder | Alcohol-related disorders - Abuse | 18 | 11 |
|  | Amphetamine (or amphetamine-like) related disorders - Abuse | 0 | 1 |
|  | Caffeine-related disorders - Intoxication | 1 | 0 |
|  | Cannabis-related disorders - Abuse | 7 | 0 |
|  | Cannabis-related disorders - Dependence | 1 | 0 |
|  | Sedative-, hypnotic-, or anxiolytic-related disorders - Abuse | 1 | 0 |

# Supplement 9: Effect sizes for between-group differences in robustness checks

| **Table S3**. *Effect sizes (Cohen’s d) for differences between healthy controls (HC), and depressed individuals without (MDD) and with comorbid anxiety disorder (MDD+ANX)* | | | | | |
| --- | --- | --- | --- | --- | --- |
|  |  |  | **Cohen’s *d*** | | |
| **Robustness check** | ***N*** |  | **HC vs. MDD** | **HC vs. MDD+ANX** | **MDD vs. MDD+ANX** |
| Male participants | 610 |  | -0.302 | 0.503 | 0.819 |
| Female participants | 1077 |  | -0.315 | 0.422 | 0.727 |
| Marburg participants | 884 |  | -0.328 | 0.507 | 0.821 |
| Münster participants | 803 |  | -0.279 | 0.349 | 0.635 |
| Acutely depressed participants | 1252 |  | -0.333 | 0.359 | 0.662 |
| Partially remitted participants | 1104 |  | -0.326 | 0.599 | 0.969 |
| Fully remitted participants | 1143 |  | -0.227 | 0.370 | 0.597 |
| Corrected for non-linear age effects | 1143 |  | -0.299 | 0.423 | 0.719 |
| Corrected for head motion | 1143 |  | -0.268 | 0.437 | 0.70 |
| Excluding connectome outliers | 1143 |  | -0.337 | 0.406 | 0.746 |
| Corrected for GMV of nodes | 1143 |  | -0.252 | 0.451 | 0.732 |
| Inclusion of subcortical nodes | 1143 |  | -0.227 | 0.231 | 0.464 |
| *Note.* *N*=Total size of the sample included in the respective analysis. GMV=Gray matter volume. | | | | | |

# Supplement 10: Networks identified at different NBS thresholds

In NBS, the choice of the primary test-statistic threshold is, to some extent, arbitrary, as it primarily determines the extent and focality of the detected network rather than its statistical significance (see Supplement 6). To demonstrate that our findings are robust across different threshold choices, we conducted additional NBS analyses using increasingly lenient primary thresholds. In our primary analysis, we chose a threshold of *t*=1.96, which corresponds to a two-tailed *p*-value of *p*<0.05 at the edge level. To examine the robustness of our findings, we additionally applied two more lenient, arbitrarily chosen thresholds (*t*=1.5 and *t*=1.0). Figure S3 illustrates the networks identified at these three different *t*-thresholds, showing the relationship between threshold selection and the resulting network characteristics.

As expected, lower thresholds yielded progressively larger and more widespread networks. At the threshold used in our primary analysis (*t*=1.96), we identified a network comprising 109 edges. When using a more lenient threshold of *t*=1.5, the network expanded to include 350 edges. At *t*=1.0, the network became even more extensive (653 edges), reflecting the detection of weaker, more diffuse connectivity differences.

Crucially, the fundamental pattern of connectivity differences was consistently detected across thresholds, despite differences in network size and density: individuals with MDD consistently showed significant hypoconnectivity (decreased number of streamlines) relative to HC, whereas individuals with MDD+ANX showed significant hyperconnectivity (increased number of streamlines) relative to HC. This pattern is evident in the violin plots on the right side of Figure S3, which display the distribution of streamline counts across the three diagnostic groups for each threshold.

Effect sizes remained in comparable ranges across all thresholds: Cohen's *d*=−0.30 to −0.41 for HC vs. MDD, *d*=0.27 to 0.42 for HC vs. MDD+ANX, and *d*=0.65 to 0.72 for MDD vs. MDD+ANX. These findings confirm that while the choice of threshold influences the extent and density of the detected network, it does not alter the core pattern of our results.

| **Figure S3.** Networks identified at different NBS thresholds. |
| --- |
| 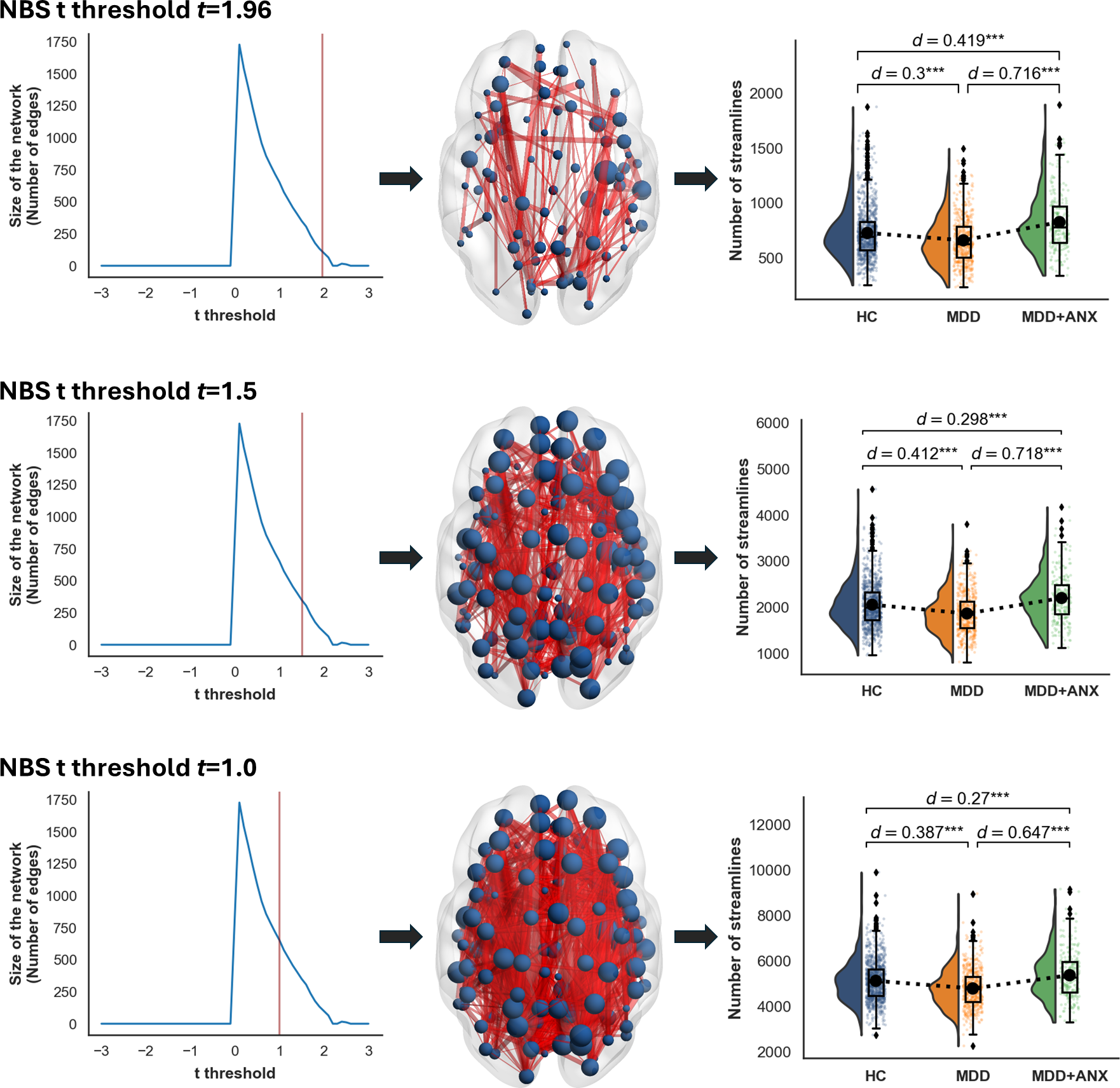 |
| *Note.* The figure displays the results of directed NBS analyses testing for the pattern MDD<HC<MDD+ANX at three different primary *t*-thresholds. The left panels show the relationship between threshold and network size (number of edges). The middle panels visualize the identified networks, with nodes representing brain regions and edges representing white matter connections showing the characteristic pattern of decreased connectivity in MDD and increased connectivity in MDD+ANX relative to HC. The right panels present violin plots illustrating the distribution of streamline counts within the identified networks across the three diagnostic groups. All analyses were corrected for age, sex, and scanner site. ****p*<0.001. |

# Supplemental References

[Andersson, J. L. R., & Sotiropoulos, S. N. (2016). An integrated approach to correction for off-resonance effects and subject movement in diffusion MR imaging. *NeuroImage*, *125*, 1063–1078. https://doi.org/10.1016/j.neuroimage.2015.10.019](https://www.zotero.org/google-docs/?uT6EGE)

[Cammoun, L., Gigandet, X., Meskaldji, D., Thiran, J. P., Sporns, O., Do, K. Q., Maeder, P., Meuli, R., & Hagmann, P. (2012). Mapping the human connectome at multiple scales with diffusion spectrum MRI. *Journal of Neuroscience Methods*, *203*(2), 386–397. https://doi.org/10.1016/j.jneumeth.2011.09.031](https://www.zotero.org/google-docs/?uT6EGE)

[Chang, L. C., Jones, D. K., & Pierpaoli, C. (2005). RESTORE: Robust estimation of tensors by outlier rejection. *Magnetic Resonance in Medicine*, *53*(5), 1088–1095. https://doi.org/10.1002/mrm.20426](https://www.zotero.org/google-docs/?uT6EGE)

[Chang, L. C., Walker, L., & Pierpaoli, C. (2012). Informed RESTORE: A method for robust estimation of diffusion tensor from low redundancy datasets in the presence of physiological noise artifacts. *Magnetic Resonance in Medicine*, *68*(5), 1654–1663. https://doi.org/10.1002/mrm.24173](https://www.zotero.org/google-docs/?uT6EGE)

[de Lange, S. C., Helwegen, K., & van den Heuvel, M. P. (2023). Structural and functional connectivity reconstruction with CATO - A Connectivity Analysis TOolbox. *NeuroImage*, *273*, 120108. https://doi.org/10.1016/j.neuroimage.2023.120108](https://www.zotero.org/google-docs/?uT6EGE)

[de Reus, M. A., & van den Heuvel, M. P. (2013). Estimating false positives and negatives in brain networks. *NeuroImage*, *70*, 402–409. https://doi.org/10.1016/j.neuroimage.2012.12.066](https://www.zotero.org/google-docs/?uT6EGE)

[Desikan, R. S., Ségonne, F., Fischl, B., Quinn, B. T., Dickerson, B. C., Blacker, D., Buckner, R. L., Dale, A. M., Maguire, R. P., Hyman, B. T., Albert, M. S., & Killiany, R. J. (2006). An automated labeling system for subdividing the human cerebral cortex on MRI scans into gyral based regions of interest. *NeuroImage*, *31*(3), 968–980. https://doi.org/10.1016/j.neuroimage.2006.01.021](https://www.zotero.org/google-docs/?uT6EGE)

[Gruber, M., Mauritz, M., Meinert, S., Grotegerd, D., Lange, S. C. de, Grumbach, P., Goltermann, J., Winter, N. R., Waltemate, L., Lemke, H., Thiel, K., Winter, A., Breuer, F., Borgers, T., Enneking, V., Klug, M., Brosch, K., Meller, T., Pfarr, J.-K., … Repple, J. (2023). Cognitive performance and brain structural connectome alterations in major depressive disorder. *Psychological Medicine*, 1–12. https://doi.org/10.1017/S0033291722004007](https://www.zotero.org/google-docs/?uT6EGE)

[Hagmann, P., Cammoun, L., Gigandet, X., Meuli, R., Honey, C. J., Van Wedeen, J., & Sporns, O. (2008). Mapping the structural core of human cerebral cortex. *PLoS Biology*, *6*(7), 1479–1493. https://doi.org/10.1371/journal.pbio.0060159](https://www.zotero.org/google-docs/?uT6EGE)

[Mori, S., & van Zijl, P. (2002). Fiber tracking: Principles and strategies—A technical review. *NMR in Biomedicine*, *15*(7–8), 468–480. https://doi.org/10.1002/NBM.781](https://www.zotero.org/google-docs/?uT6EGE)

[*Reference Manual for NBS Connectome (v1.2)*. (2012, December).](https://www.zotero.org/google-docs/?uT6EGE)

[Repple, J., Gruber, M., Mauritz, M., de Lange, S. C., Winter, N. R., Opel, N., Goltermann, J., Meinert, S., Grotegerd, D., Leehr, E. J., Enneking, V., Borgers, T., Klug, M., Lemke, H., Waltemate, L., Thiel, K., Winter, A., Breuer, F., Grumbach, P., … Dannlowski, U. (2023). Shared and Specific Patterns of Structural Brain Connectivity Across Affective and Psychotic Disorders. *Biological Psychiatry*, *93*(2), 178–186. https://doi.org/10.1016/j.biopsych.2022.05.031](https://www.zotero.org/google-docs/?uT6EGE)

[Repple, J., Mauritz, M., Meinert, S., de Lange, S. C., Grotegerd, D., Opel, N., Redlich, R., Hahn, T., Förster, K., Leehr, E. J., Winter, N., Goltermann, J., Enneking, V., Fingas, S. M., Lemke, H., Waltemate, L., Nenadic, I., Krug, A., Brosch, K., … van den Heuvel, M. P. (2020). Severity of current depression and remission status are associated with structural connectome alterations in major depressive disorder. *Molecular Psychiatry*, *25*(7), 1550–1558. https://doi.org/10.1038/s41380-019-0603-1](https://www.zotero.org/google-docs/?uT6EGE)

[Sarwar, T., Ramamohanarao, K., & Zalesky, A. (2019). Mapping connectomes with diffusion MRI: deterministic or probabilistic tractography? *Magnetic Resonance in Medicine*, *81*(2), 1368–1384. https://doi.org/10.1002/mrm.27471](https://www.zotero.org/google-docs/?uT6EGE)

[Van Den Heuvel, M. P., Scholtens, L. H., Van Der Burgh, H. K., Agosta, F., Alloza, C., Arango, C., Auyeung, B., Baron-Cohen, S., Basaia, S., Benders, M. J. N. L., Beyer, F., Booij, L., Braun, K. P. J., Filho, G. B., Cahn, W., Cannon, D. M., Chaim-Avancini, T. M., Chan, S. S. M., Chen, E. Y. H., … De Lange, S. C. (2019). 10kin1day: A bottom-up neuroimaging initiative. *Frontiers in Neurology*, *10*(MAY), 425. https://doi.org/10.3389/fneur.2019.00425](https://www.zotero.org/google-docs/?uT6EGE)

[Van Den Heuvel, M. P., Sporns, O., Collin, G., Scheewe, T., Mandl, R. C. W., Cahn, W., Goni, J., Pol, H. E. H., & Kahn, R. S. (2013). Abnormal rich club organization and functional brain dynamics in schizophrenia. *JAMA Psychiatry*, *70*(8), 783–792. https://doi.org/10.1001/jamapsychiatry.2013.1328](https://www.zotero.org/google-docs/?uT6EGE)

[Woolrich, M. W., Jbabdi, S., Patenaude, B., Chappell, M., Makni, S., Behrens, T., Beckmann, C., Jenkinson, M., & Smith, S. M. (2009). Bayesian analysis of neuroimaging data in FSL. *NeuroImage*, *45*(1 Suppl).](https://www.zotero.org/google-docs/?uT6EGE)

[Zalesky, A., Fornito, A., & Bullmore, E. T. (2010). Network-based statistic: Identifying differences in brain networks. *NeuroImage*, *53*(4), 1197–1207. https://doi.org/10.1016/j.neuroimage.2010.06.041](https://www.zotero.org/google-docs/?uT6EGE)

[Zalesky, A., Fornito, A., Cocchi, L., Gollo, L. L., van den Heuvel, M. P., & Breakspear, M. (2016). Connectome sensitivity or specificity: Which is more important? *NeuroImage*, *142*, 407–420. https://doi.org/10.1016/J.NEUROIMAGE.2016.06.035](https://www.zotero.org/google-docs/?uT6EGE)
